# Computer Vision Meets Image Processing and UAS PhotoGrammetric Data Integration: From HBIM to the eXtended Reality Project of Arco della Pace in Milan and Its Decorative Complexity

**DOI:** 10.3390/jimaging7070118

**Published:** 2021-07-16

**Authors:** Fabrizio Banfi, Alessandro Mandelli

**Affiliations:** Architecture, Built Environment and Construction Engineering (ABC) Department, Politecnico di Milano, 20133 Milano, Italy; alessandro.mandelli@polimi.it

**Keywords:** Unmanned Aerial System (UAS), heritage documentation, photogrammetry, 3D modelling, eXtended Reality (XR), virtual museums, computer vision

## Abstract

This study aims to enrich the knowledge of the monument Arco della Pace in Milan, surveying and modelling the sculpture that crowns the upper part of the building. The statues and the decorative apparatus are recorded with the photogrammetric technique using both a terrestrial camera and an Unmanned Aerial Vehicle (UAV). Research results and performance are oriented to improve computer vision and image processing integration with Unmanned Aerial System (UAS) photogrammetric data to enhance interactivity and information sharing between user and digital heritage models. The vast number of images captured from terrestrial and aerial photogrammetry will also permit to use of the Historic Building Information Modelling (HBIM) model in an eXtended Reality (XR) project developed ad-hoc, allowing different types of users (professionals, non-expert users, virtual tourists, and students) and devices (mobile phones, tablets, PCs, VR headsets) to access details and information that are not visible from the ground.

## 1. Introduction

In recent years, drone photogrammetry has become part of the daily life of many professionals in many different sectors. The main application fields are cultural heritage [1], archaeology [2], geology [3], technical and thematic cartography [4], crime and accident scene [5], human bodies survey [6], and rapid survey photogrammetry from video [7]. The starting data is always a set of photographs and therefore a set of two-dimensional digital images that are processed by the software to extract three-dimensional data. The accuracy of the work is the main advantage of photogrammetry with a drone [8,9,10]. Manually measuring sites can lead to several (human) errors on the part of professionals. On the other hand, drones avoid these errors and allow us to obtain accurate data, allowing surveys to be carried out with greater accuracy, significantly increasing safety at work and allowing us to reach places that are difficult for humans to access.

Thus, the Architecture, Engineering and Construction (AEC) sector is increasingly concerned with advanced simulation to create objects that behave and look as authentic as possible.

Furthermore, in the digital cultural heritage (DCH) domain, interest is growing in describing the geometry and behaviour of 3D objects through a scan-to-BIM process [11,12]. Accordingly, the integration of image processing, computer vision and 3D modelling have become significantly more useful in architecture, engineering, advanced prototyping, healthcare, and design [13,14,15,16]. In this context, the most important new movement in graphics is increasing concern for modelling objects, allowing professionals to increase the graphic value and information of heritage buildings, monuments, and archaeological sites.

For these reasons, the authors propose a scientific method based on digital photogrammetry, laser scanning and Building Information Modelling for Heritage artefact (HBIM), where it has been possible to go beyond the main 3D representation techniques, obtaining digital representations capable of communicating high Levels of Detail (LOD) and Levels of Information (LOI). On the other hand, the digitisation process of a historic building and its morphological and typological complexities requires high skill and knowledge of professional software capable of transforming simple images and 3D scans into informative models. The study here follows the workflow from the 3D photogrammetric survey phase to the digital delivery and presentation of the results through an eXtended Reality (XR) project that allows many users to employ different devices to access data and information directly accessible in other ways. Regarding this aspect, many techniques can be used to acquire the shape of ornaments and statues, but doubtless the most efficient way is using an Unmanned Aerial System (UAS). A piloted aircraft lets the surveyor get very close to the objects without wasting money hiring cranes or platforms to reach the top of the arch.

## 2. Motivation and Main Contributions

The authors’ research in recent years has focused on improving the scan-to-BIM process of historic buildings, proposing and developing methods capable of automating the generative process of the model and maintaining high LODs and LOIs. On the other hand, in recent years, the construction sector has witnessed an epochal change that has led to re-engineering of the daily practices of architects, engineers, and archaeologists. Thanks to the benefits found in 3D surveying, digital photogrammetry (terrestrial and aerial) and the advent of XR development platforms, it has been possible to improve the use of scan-to-BIM models for interactive environments, increasing information sharing and reaching time for a wider audience such as students and virtual tourists. For this reason, this article proposes a method capable of enhancing the use of HBIM models to develop immersive XR environments, where the user can interact with new levels of interactivity.

The research method is based on four main research phases:

Data collection: the data collection and analysis phase envisaged primary and secondary data sources. Authors digitised the Arco della Pace monument through 3D survey techniques such as terrestrial, aerial photogrammetry and laser scanning. The main outputs of this phase are point clouds, orthophotos and mesh textured models both for the architectural elements and for the decorative apparatus composed of low reliefs and sculptures. These outputs are considered primary data sources. Secondary data sources, on the other hand, include different types of analyses and studies. The latter were conducted on various historical texts to better understand the construction technique of the monument, its historical and cultural background and the artistic values of the decorative apparatus.

Scan-to-BIM process: the digitisation process of the monument is described to show how many primary data sources have been processed sustainably, transformed into digital models capable of interacting like the latest generation applications in the construction sector. Consequently, the process of transformation and orientation of the digital models had to be based on a scientific study capable of considering different levels of interoperability of models from images, point clouds and textured mesh models.

Information mapping: once the various 3D objects have been created, a phase of mapping information is undertaken both of a visual and graphic nature (physical and mechanical characteristics of the materials, masonry stratigraphy, historical phases) and of a historical and cultural character through texts and descriptions. This phase allowed the authors to move from simple static models to objects capable of communicating different types of information.

Information sharing: finally, tests and studies were conducted to improve the models’ interactivity level. Thanks to the definition of sustainable digital workflow, it has been possible to transform static models into interactive virtual objects capable of maintaining the quality of the virtual experience. Finally, the most advanced forms of virtual and augmented reality have been tested in order to reach different types of users and the latest generation devices.

The article is structured as follows:

- a first part is dedicated to state of the art, divided in turn into a synthetic framework oriented towards HBIM and the forms of XR for the built heritage and a framework on aerial photogrammetry and its regulatory context;
- a description of the case study both from a historical-cultural point of view and from a geographical and regulatory point of view;
- the description of the method that has enabled the transformation of simple points and mesh models from 3D survey and digital photogrammetry into complex digital models (NURBS and HBIM) and XR projects with different levels of interactivity, information and immersion;
- A concluding part dedicated to a discussion of the results through a holistic approach and related conclusions.

## 3. State of the Art

### 3.1. State of the Art about Heritage Building Information Modelling Oriented to eXtended Reality (XR)

The monument of the Arco della Pace presents numerous complexities, both from a constructive point of view and from an architectural and decorative point of view. The digitalisation process consequently required high knowledge of 3D modelling and BIM to transmit geometric, metric, and informative values at the same time. As anticipated in the previous paragraphs, the urgency of communicating information to different types of users required the study and integration of varying representation techniques, from descriptive geometry to the scan-to-BIM process [17,18,19,20,21,22]. The latter, in recent years, has shown how, through the application of specific scan-to-BIM requirements and grades of generation (GOGs) [23], it is possible to go beyond the modelling of parametric objects included in the default libraries of the main BIM applications such as Autodesk Revit and Graphisoft Archicad [24,25,26]. As known, such applications allow users to add information to three-dimensional objects, which in turn represent the architectural and structural components of buildings [27,28,29]. In the early 90s, BIM was developed for the management of new buildings, where the use of object libraries corresponding to early walls of geometric irregularities, including a wide range of choice between standard objects such as doors, windows, floors, false ceilings, and furnishings, allowed the user to faithfully represent his project and associate it with information of a physical, mechanical nature, etc. Consequently, the entire construction sector has been transformed in its daily practices, where architects and engineers have had to face a significant change, passing from the first to the second digital era. The transition almost entirely saw the abandonment of representation and manual drawing favouring CAD vector design and then subsequently BIM [30,31,32].

On the other hand, the benefits brought about by this new method and tools laid the foundations for the definition of new fields of applications based on digital models, such as restoration, energy analysis, finite element analysis, estimation, construction site and many others [33,34,35,36]. Consequently, the urgency of orienting the digitisation project of the existing building involved integrating 3D survey techniques with BIM. In particular, thanks to laser scanning and digital photogrammetry, it was possible to lay the appropriate foundations to represent existing buildings correctly [37,38,39]. The last decades have been characterised by the definition of new innovative methods, guidelines and standards that have defined this new field of application. Furthermore, interesting research in representation, geomatics, and restoration has proposed methods capable of speeding up the digitisation process of complex elements of historic buildings [21,40,41,42,43]. In this particular context, as is well known, complex vaults systems, irregular walls and decorative devices require advanced modelling techniques, where BIM modelling tools do not allow for fast and faithful representation of the building surveyed [44,45,46].

For this reason, the authors’ research in recent years has focused on the definition of workflows capable of representing historic buildings characterised by high levels of detail and information. The definition of these methods also required an in-depth study of computer programming, through which it was possible to develop not only sustainable application methods but digital tools capable of improving the level of automation of the scan-to-BIM process [47,48,49]. Thanks to previous studies, the case study of the Arco della Pace monument, its architectural, cultural, historical, and artistic complexities, have laid the foundations for proposing a method capable of going beyond what is defined today in the international panorama of digital cultural heritage [50,51,52].

### 3.2. State of the Art about Regulation for Flying Drones in Italy and Europe

In the last decades, the use of Unmanned Aerial Vehicles in the construction and architecture field underwent significant development thanks to the increasing ease of use in piloting the vehicles and thanks to the better quality of the photographic sensors. UAVs are employed in different scenarios in the AEC (Architecture, Engineering and Construction) sector; high-resolution photographic sensors, IR sensors and thermal ones are widely used for monitoring and inspection purposes [53]. UAVs let the operators get close to the structures and buildings, preventing cranes and safeguarding the workers. Nevertheless, the time required for monitoring and inspection activities is shorter than using other terrestrial vehicles.

In the same way, the high number of images that can be acquired during a flight let the operators use the images for photogrammetric projects. In these cases, the flight had to be planned carefully to ensure the minimum overlap among the images and to avoid lack of data at the end of the elaboration. This technology permits us to reach and acquire parts of the buildings and structures that are unreachable with other instruments or require significant efforts to be mapped.

In the Cultural Heritage field, buildings are often decorated with complex friezes and ornaments that produce shaded areas when they are surveyed from ground level, both with laser scanning techniques and photogrammetry. For these reasons, in the last years, different operators decided to adopt UAVs in their daily working activities. Therefore, the regulatory bodies were forced to emanate rules to prevent accidents and interferences with regular commercial and touristic air traffic. In Italy, the first regulation concerning UAVs was enacted by the National Authority for Civil Aviation (ENAC, Ente Nazionale per l’Aviazione Civile) on the 16th of December 2013, then many editions and amendments were issued up to the present today. On the 31st of December 2020, the European regulation became effective, significantly changing many articles of the previous Italian regulation [54].

At the time of the UAV survey of Arco della Pace in Milan, November 2020, and now at the time this article is being written, the Italian and European regulations are still effective until the 1st of January 2023. The delays of the emanation of a unique and clear law are due to the COVID-19 pandemic; in fact, the transition process and alignment to the new regulation by the producers of Unmanned Aerial System (UAS) should have been completed by the 1st of January 2021. On this date, in Italy, the first edition of the UAS-IT regulation that transposes the implementing UE regulation 2019/947 concerning rules and procedures to fly Unmanned Aerial Vehicles was enacted (Table 1).

This situation generated some difficulties in understanding which rules were effective at the time of the survey and how best to acquire permission to perform the 3D survey of the monument. The “No of Pages” column of the table above shows that the regulation and amendments changed over the years by adding, removing or slightly modifying the contents of single articles.

#### 3.2.1. Italian Regulation UAV

Looking at the Italian regulation, it is interesting to analyse the changes between the first, second and third edition of the Italian Regulation (UAV) and the first edition of the Italian Regulation (UAS-IT). As it is clear from the title, the last Italian regulation is now aligned with the European one, focusing attention on the system: vehicle and radio control station. Now the acronym used is UAS and no more UAV. Moreover, it is interesting to notice a difference of more than 16 pages between the first and second editions of the Italian regulation. The last edition of 04 January 2021 has 17 pages less than the second edition of the 16 July 2015. The first edition (16 December 2013) of the Italian regulation is divided in 6 sections that include 26 articles. This version of the regulation is composed mainly of definitions and references to other rules and laws concerning airworthiness. Section 2 and Section 3 differentiate between UAVs according to their weight, and there are different procedures for flying UAVs if they weigh more or less than 25 kg. There is general information about UAVs weighing less than 2 kg that may follow simplified procedures to get permission to fly. The procedures to acquire permission are not clear at all. In this case, general advice is provided to contact the ENAC body via e-mail and ask permission to fly by describing the pilot’s activities during the flight operations. The article regarding the pilot is unclear; in fact, it is asked that he/she holds a civil or sport flight licence and he/she must know the air rules and must have completed a training period at unclearly defined companies. Two different entities are identified in the regulation: the operator is the owner of the UAV and is responsible for the maintenance of documentation and the UAV itself, and the pilot is responsible for flight operations. A section is devoted to using UAVs for recreational purposes. Presently, there is just one article stating that UAVs can be used for recreational purposes in specified flight fields without acquiring a flight licence and without asking permission from the ENAC.

The second edition (16 July 2015) adds two sections to the previous one, namely the rules for using the air space and general rules for flying UAVs. This version of the regulation introduces some other significant changes: (i) the difference between critical and non-critical operations is addressed; (ii) the difference between specialised and non-specialised operations is defined; (iii) the procedures for flying UAVs weighing less than 2 kg are well described; and (iv) the definitions of Visual Line Of Sight (VLOS), eXtended Visual Line Of Sight (EVLOS) and Beyond Visual Line Of Sight (BVLOS) are introduced. According to this regulation, specialised operations provide a paid service, such as video or photo recording, surveillance, environmental or industrial monitoring, agriculture services, and photogrammetry. All other activities that do not consider a payload are classified as not specialised and are considered recreational. The specialised non-critical operations are always performed in VLOS, i.e., in constant eye contact with the UAVs, far from crowds, traffic, urban areas, infrastructures, and industrial plans. In these cases, the procedures to acquire permission are much more simplified than in critical operations.

Moreover, the activities performed with UAVs weighing less than 2 kg are always considered not critical. Following the evolution of the market of consumer UAVs, and namely with the presentation of the DJI Spark, the Italian Regulation added an article regarding vehicles weighing less than 300 g. The UAVs falling in this range of weight and equipped with guard propellers did not require a flight licence or permission issued by ENAC.

In the third edition (11 November 2019), some specifications are added. In the third edition of the regulation, exams are differentiated according to the kind of operations to be conducted. In the case of non-critical operations, the certificate was issued after passing an online test, but on the other hand, to acquire the certificate for critical operations, a practical exam was needed. The article regarding UAVs weighing less than 300 g was revised: the limit is now fixed to 250 g, and at least a theoretical exam is needed to fly these lightweight UAVs. This constraint was introduced because of the large consumer market of small UAVs equipped with high-resolution camera sensors. In fact, problems started to arise linked with public security and privacy since everyone older than 18 years could have bought a UAV in a supermarket and started to fly almost everywhere without knowing the basic rules of flight. In the third edition, the minimum age to drive a UAV is lowered from 18 to 16 years. Regardless of the weight or the operation, specialised or recreational, every kind of UAV must be registered in a national online database, and a QR-code must be applied to the UAV itself. The UAV operator performs the registration that must also indicate the personal data of the pilot and its certificate. The online database that manages all the activities in the Italian airspace is www.d-flight.it/new_portal (accesed on 14 July 2021) [55].

#### 3.2.2. Italian Regulation UAS-IT and European Regulation

The first edition of the new UAS-IT regulation issued on the 4 January 2021 changes the previous regulations’ vision and structure completely. This last regulation receives all the indications of the European one and disciplines the aspects that rely on the competence of the state member.

The UAS-IT regulation currently only has 5 sections, 20 pages, and 31 articles. It is shorter than the first regulation of 2013. Unfortunately, this contraction in length implies more difficulties in understanding the global view of the regulation. In fact, there are legal references to 14 different documents, (Regolamento UAS-IT, Codice della Navigazione, Regolamento (UE) n. 2018/1139 “Regolamento Basico”, Regolamento (UE) n. 2019/947, Regolamento (UE) n. 2019/945) that a UAV operator should know to understand the regulations completely. In place of the weight subdivision, the concepts of: (i) Open, Specialised and Certified Categories, (ii) class identification label, (iii) Specific Assurance and Integrity Level (SAIL) are introduced.

The Open category is a category of UAS operation that, considering the risks involved, does not require prior authorisation by the competent authority nor a declaration by the UAS operator before the operation takes place. The Specific category is a category of UAS operation that, considering the risks involved, requires authorisation by the competent authority before the operation takes place, considering the mitigation measures identified in an operational risk assessment, except for specific standard scenarios where a declaration by the operator is sufficient. The Certified category is a category of UAS operation that, considering the risks involved, requires the certification of the UAS, a licensed remote pilot and an operator approved by the competent authority to ensure an appropriate level of safety.

**Figure 1:**
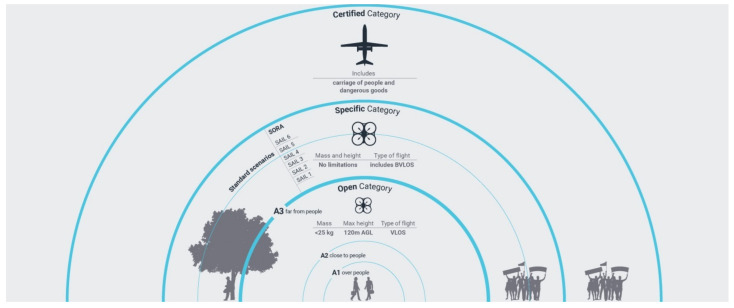
Categories Scheme, https://www.easa.europa.eu/document-library/easy-access-rules/easy-access-rules-unmanned-aircraft-systems-regulation-eu (accessed on 14 July 2021).

The Open category is itself subdivided in 3 sub-categories A1, A2, and A3, which may be summarised as follows (Figure 1):

- A1: fly over people but not over assemblies of people;
- A2: fly close to people;
- A3: fly far from people.

Each sub-category comes with its own sets of requirements. Therefore, it is important to identify which rules apply and the type of training needed in the Open category. Then, a UAV with the proper class identification label (C0, C1, C2, C3, C4) must be chosen (Table 2). Today, not even one UAV on the market has a classification label, so until the 1st of January 2023, the identification label is substituted by weight classes.

If the activities do not fall under the Open category, the operator needs an operational authorisation from the National Aviation Authority. In this category, a risk assessment is needed, and there are six different levels of risk identified by roman numbers, with each level described inside the Joint Authorities for Rulemaking of Unmanned Systems (JARUS) guidelines on Specific Operations Risk Assessment (SORA). Working with UAVs is quite complex because the rules are constantly evolving, and in the last two years, the concept of regulation changed completely, passing from weight and type of operation classification to a classification based on the risk of activities. In this scenario, it was not easy to approach the survey of the Arco della Pace because it meant flying in the city centre of Milan, very close to people, traffic and inside a no-fly zone. Lastly, it must be considered that if these operations are conducted without respecting the law, the penalties are the same as the Civil Aviation Code, starting from tens of thousands of euros.

## 4. The Research Case Study: Historical and Cultural Background, Monument Location and Flight Restrictions

### 4.1. The Arco della Pace in Milan: Origins and History of the Arco

**Figure 2:**
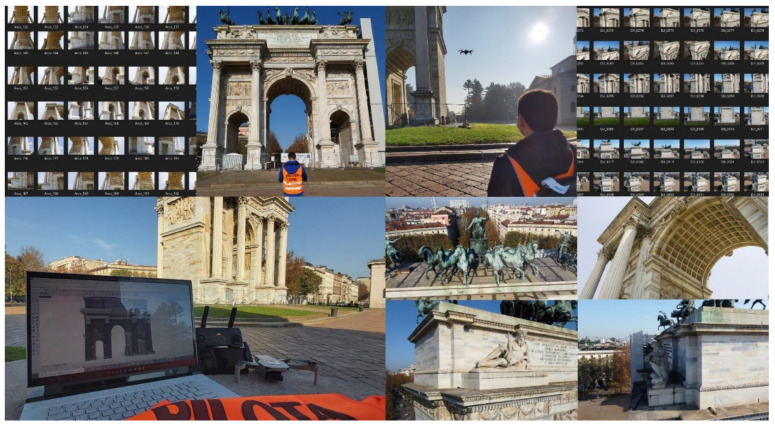
The research case study: images from terrestrial (**left**) and UAV survey (**right**).

The Arco della Pace can be considered as the only example of a triumphal and monumental entrance to Milan, with its symbolic and commemorative presence. The arch is in the place of arrival of Corso Sempione in connection with Paris, or at the Porta Sempione, which for decades was the entrance to the city of Milan (Figure 2). The arch assumed enormous urban importance after the demolition, in 1801, of the star of the sixteenth- and seventeenth-century fortifications, when a new access road was traced and a new door was built on the axis of the Castello Sforzesco. The urban importance of the Porta del Sempione becomes substantial when one thinks that before this, the roads connecting Milan with the territory to the north-west avoided the Castle and penetrated the city, passing through openings at the points of union between the Castle and the Spanish walls called “portelli”. As a result, the place mutates in meaning: from a barrier, it becomes a passage and therefore a point of attraction towards the city centre, becoming, during the Napoleonic Empire, the main entrance to Milan.

On the 15th of May 1796, with the entry into Milan of the Napoleonic troops, and with the founding of the Cisalpine Republic first and then of the Kingdom of Italy, a positive period of effective possibilities began for the building and urban reorganisation of the city. In 1806, a “Commission of Architecture and Fine Arts” was set up, delegated to indicate the general directions for the arrangement of the city public spaces. The commission, composed of Bossi, Canonica, Appiani, Podestà, Brivio, Cagnola and Zanoja, set up an extensive urban restructuring program, with references to the French tradition of embellishment. However, innovative aspects were also introduced, both in the overall vision of the settlement and in the variation of the structure in the urban fabric. The “Plan des artistes” of 1793 and the “Plan of the embellishment” for 1798 in Paris were the reference planes.

Among the interventions suggested by the commission were an arch to be erected at the Sempione barrier and the completion of the Eastern Gate, the arrangement of the Porta Vercellina, the decoration of the Forum barracks, the decoration of the Amphitheater, the construction of a bridge between the district of S. Andrea coni Boschetti and the Collegio Elvetico, and the decoration of the Palazzo dei Giardini Pubblici.

**Figure 3:**
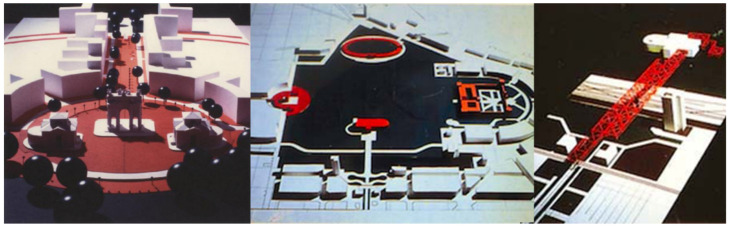
Vittoriano Vigano’s plan: model of Piazza Sempione for the enhancement plan of Parco Sempione and its monuments (1996). (Location: Milan (MI), La Triennale di Milano Foundation, Photo Archive of the Milan Triennale, TRN_XIX_03_0145).

One of the most recent enhancement plans for the Sempione Park and all its monuments is the work of Vittoriano Viganò in 1954. It was conceived as a recovery plan for a part of Milan historically homogeneous in its monumental identity. Due to the increase in road traffic and disinterest in the post-war period, it entered a state of neglect and decay. The plan conceived by Viganò is based on an idea of an urban relaunch primarily involving this large area and its identification, in the sense of open, public, and recognisable space, which can be enjoyed in various areas (Figure 3). The plan covers an area of approximately one million square meters. The Sempione system is an urban and architectural complex, rediscovering its own identity, connections, and entirety, that will come to be born as a new major attraction in Milan.

Its eventual unification and modernisation could contribute to the functional revival of the monuments and transform the park heritage from an interval of the urban continuum into a homogeneous part of characteristic significance, capable of extending the historic core towards the north-west in a unique way. The start-up of the plan was set up in parts, and Piazza Sempione corresponds with what is defined as the first intervention unit (1980–1986). This intervention is followed by others that concern the whole system of the park up to Piazza Castello. Since the plan is very ambitious, it sparked various “appetites” that tried to oppose the “Franciscan force of non-speculable space, his life is difficult, and the management was slow and laborious”.

Suffice it to say that the plan, introduced in 1955, only became operational in the 1990s. The municipal administration decided to undertake a general restoration of the park and Piazza Sempione with the Arco della Pace. The intervention involved a new fence for the park, the renewal of the roads, and park botanical and floristic renewal. One of the intentions of the project was to bury all the driveways to incorporate Piazza Castello and the first stretch of Corso Sempione.

### 4.2. Ornamental and Decorative Elements

**Figure 4:**
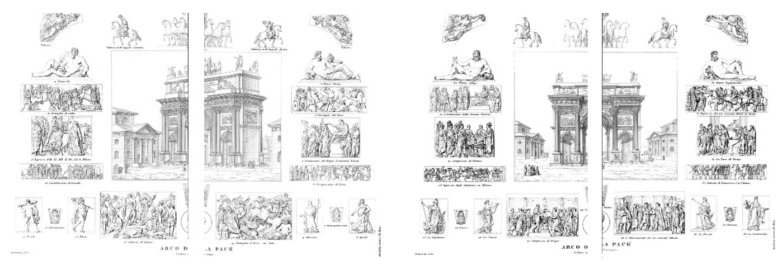
Historical reports: the ornaments of the Arco della Pace. Overall table of the elements relating to the front of the monument towards the Castello Sforzesco (**left**) and Corso Sempione (**right**), taken from the publication edited by G. Reina and published in 1856. Di Baio historic archive. (Giani, G., L’Arco della Pace di Milano, Di Baio Editore, Milano, 1988).

The Arco della Pace is rich in decorative and ornamental elements, which underwent some retouching to represent the new Austrian course (Figure 4). However, the eight allegorical bas-reliefs on the pedestals were already present at the time of the new plan. The will of the central Congregation was to remind its citizens of the achievements that contributed to the Kingdom’s birth. However, since the historical events that they wanted to represent were too abundant to affect a triumphal arch, only those relating to the most important events were chosen. Allegorical and allusive figures were then chosen to evoke “the beautiful arts, the fertility of the Lombard soil, the historical events” that were most significant. To these are added other events, including the Congress of Prague, the Meeting of the Three Great Allies and other war enterprises, thanks to which the much-desired peace was obtained. Passage of the Rhine, Capitulation of Dresden, Battle of Ar-cis-sur-Aube, Occupation of Lyon, the Battle of Paris and, finally, the triumphal entry of the three monarchs into the city of the French Empire. These findings were the works of many sculptors including Camillo Pacetti, Luigi Acquisti, and Pompeo Marchesi. In addition to the military enterprises, the political operations that made the Peace of Paris and the Congress of Vienna official were also mentioned.

### 4.3. Monument Location and Flight Restrictions

**Figure 5:**
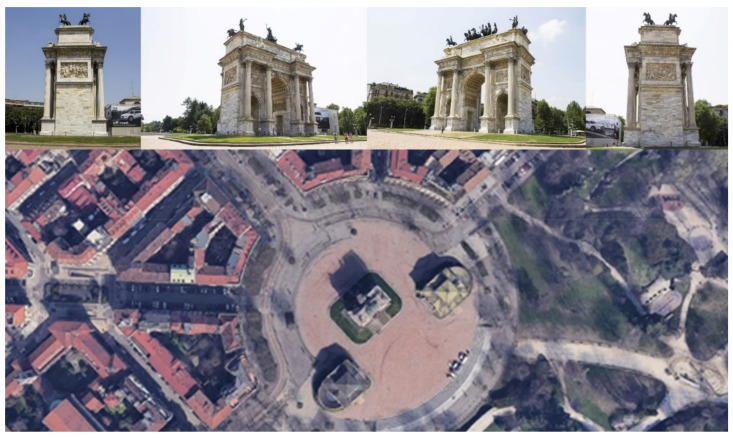
Google Map image centred on the monument.

As anticipated, the monument is in the city centre of Milan in the centre of Piazza Sempione, an important city hub (Figure 5).

Here ends Parco Sempione, one of the biggest parks of Milan. Local people and tourists always crowd this place; moreover, Piazza Sempione is half surrounded by jammed streets and punctuated by dehors of the adjacent bars and restaurants.

**Figure 6:**
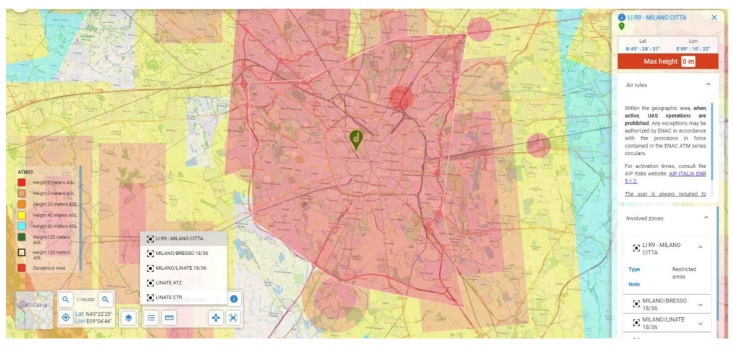
D-Flight Map image centred on the monument, with restriction areas highlighted. Each colour refers to a height limit for flight above the city of Milan. In the red areas, flight is prohibited. Source: www.d-flight.it/newportal, accessed on 14 July 2021).

Considering the regulations, both Italian and European, flight in such places is forbidden because it will interfere with public activities around the monument. Moreover, Italian regulations on airspaces subdivide the national ground into different zones with special rules concerning flying with manned or unmanned vehicles. Consulting the aeronautical maps provided by the Italian online database for UAVs, it appears that the area of the survey falls into four different restriction areas where flight is forbidden to anyone, mainly for security reasons (Figure 6):

- LI-R9 Milano-Città;
- Milano/Bresso 18/36;
- Milano/Linate 18/36;
- Linate Aerodrome Traffic Zone (ATZ);
- Linate Control Traffic Region (CTR).

The city authorities can obtain temporary permission by submitting all the necessary documents and a high detailed relation that describes the activity, the timetable of the flights, the risk assessment and the precautions taken to decrease the level of the risk. The authors, both holding a piloting license, provided the material mentioned above to the prefecture of Milan that has the faculty to issue the permission, then permission also had to be approved by the ENAC. Even if the prefecture issues permission, the Authority can revoke it. A month after submitting the request, the survey activities described in relation received a positive judgment from the two authorities. The pilots considered a sufficient buffer area around the monument, and they chose a date that fell in the lockdown period linked with the COVID-19 pandemic. Therefore, all the shops’ restoration activities were closed, and there were no crowds around the monument due to the prohibition on staying in public spaces without a proven reason.

## 5. Material and Methods: From Geometrical Surveys to HBIM, Virtual Museums and eXtended Reality

**Figure 7:**
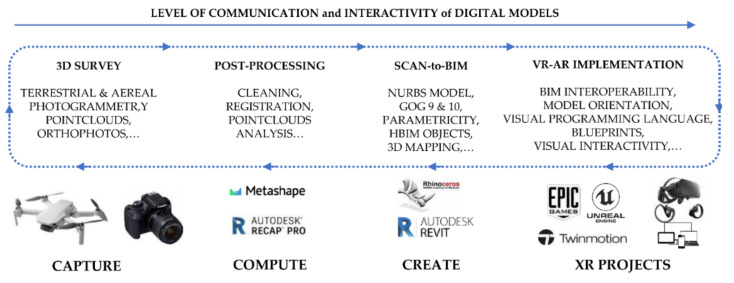
The digital workflow applied to the research case study.

The method proposed in this paragraph has been structured in an attempt to outline an operational workflow that is as sustainable as possible (Figure 7). The key factors for improving the scan-to-BIM-to-XR process of the monument were:

- Integration of aerial photogrammetry in the building digitisation process to complete the textured digital model;
- 3D mapping able to be automatically recognised through the real-time synchronisation of multiple environments, from NURBS modelling software and BIM platforms to XR development platforms;
- Interoperability and synchronisation of digital models in various environments; automatic recognition and real-time synchronisation of digital models through the main 3D exchange formats (open source and not) such as the 3DM, DWG, RVT, FBX, OBJ;
- The interactivity of XR projects; through IT development based on VPLs and Blueprints, it has been possible to create interactive virtual objects capable of interacting with all user inputs on different kinds of devices (tablets, laptops, PCs, and mobile phones).

### 5.1. UAV Photogrammetric Survey

Since the Italian regulation about airspaces and the upcoming European law consider special rules for UAVs weighing less than 250 g, it was decided to use a UAV with this peculiar characteristic. Moreover, the Italian law obliges the drone pilot to install guard propellers to use the drone in a public space, such as Piazza Sempione, where the monument is. For these reasons, the survey team employed a DJI Mavic Mini for the photogrammetric survey (Table 3). The photogrammetric survey was sided by topographic measurements performed with the Leica TS12 (Leica Geosystems AG, Heerbrugg, Switzerland). A simple network of four points was created around the monument, two vertices are linked through the open passing arch in the centre. From the station points, some natural points were measured on three sides of the monument (one side is covered from the base to the top with scaffolding for restoration activities) to check the correctness of the photogrammetric elaborations. The aim of the survey was to collect enough data to represent the arch by means of a 3D model at a 1:50 drawing scale.

Considering the DJI Mavic Mini specification listed above, it is possible to calculate the mean distance of acquisition to get the desired resolution. It was decided to assume the “plotting error” (p.e.) as the parameter to calculate the distance of acquisition. This value derives from the cartography field and is related to the precision of a map, and it is conventionally assumed to be equal to 0.2 mm. The p.e. obviously changes in relation to the scale of the map; 0.2 mm must be multiplied by the scale factor. Consequently, the p.e. at a 1:50 scale is equal to 1 cm. Conventionally in cartography, the sampling measurements tolerance is assumed equal to 2 times the p.e. Moving from the 2D cartography field to the photogrammetric 3D domain, it was decided to impose a Ground Sampling Distance (GSD) equal to the p.e. at 1:50.

p.e._1:50_ = 0.2 mm × 50 = 1 cm = GSD_1:50_ (1)

The distance of acquisition to reach at least this value at the end of the survey is computed with the equation:

c:D = px:GSD

4.49 mm:D = 0.00162 mm:10 mm(2)

D = 27 m

where: c = focal length, D = distance of acquisition, px = pixel size, GSD = Ground Sampling Distance.

Consequently, 27 m represents the theoretical value considering an ideal condition with the camera placed on a tripod without external interferences. The practical activity suggests, even in good conditions, halving the distance from the surveyed object to avoid poor data at the end of the survey, and in this case, the authors decided to fly at a mean distance of 10 m from the monument. Therefore, the number of images increased significantly. From this, 945 images at 12 MPixels, the maximum resolution of the camera, were collected in JPG format during three flights to cover all the facades of the monument, the decorative apparatus and the statues that crown the top of the arch. The flights were performed in manual mode, frequently changing the orientation of the camera gimbal to capture the complexity of the shapes from different views and to cover all the possible shadow areas on the monument. As much as possible, the flights followed regular paths, performing vertical strips all around the building. The vertical (longitudinal) overlap of the images was controlled by setting the auto interval acquisition of the images equal to 2 s, and the side (transversal) overlap was valued directly on the screen by the video operator of the drone.

### 5.2. Terrestrial Photogrammetric Survey

Due to the scaffolding from the base to the top on the east side of the monument, it was decided to merge the data of the photogrammetric flight with the photogrammetric terrestrial data acquired in 2019 when the scaffolding was not in place.

That survey was performed to produce a parametric model with Rhinoceros, starting from the dense point clouds computed at the end of the photogrammetric process. The same procedure was adopted on that occasion, and the photogrammetric survey was followed by a topographic campaign. The camera used was a Canon EOS 1100D (Canon Inc. Ōta, Tokyo, Japan) coupled with an 18 mm lens (Table 4).

The distance of acquisition was calculated as before to produce a dense point cloud with an accuracy of 1 cm.

### 5.3. UAV Data Elaboration

The UAV data were elaborated following a pyramidal schema, from general to particular, using Agisoft Metashape Pro (version 1.7.2 build 12070). Firstly, all the 945 images were imported into the software; the GPS information, namely the position of the drone during the acquisition of each single image, and the orientation of the camera were removed before the alignment phase. The elaboration considered two phases: the first was useful to elaborate all the images simultaneously and find the 3D dense point cloud of the top architectonical elements of the arch, which were not visible form the terrestrial photogrammetric survey. The second phase considered only the decorations, the statues, and the bass reliefs. Starting from the results of the first alignment, the bounding box was then limited around each decorative element, and 3D mesh reconstruction was performed to access their high-resolution models separately from the architecture.

#### 5.3.1. The Building

**Figure 8:**
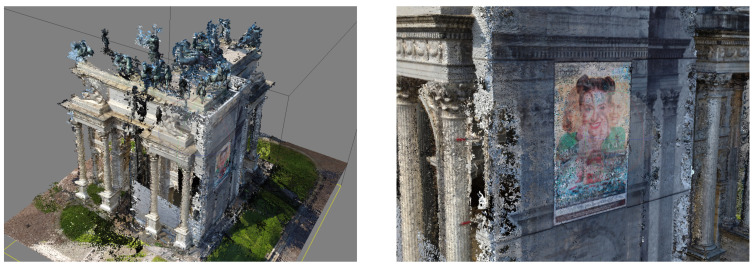
On the left, the misalignment of the images; on the right, the maxi-screen that prevented the correct alignment of the images.

Immediately, some problems arose. In fact, even if the software said that all the images were “correctly” aligned, it appeared clear that there were some errors in the geometrical reconstruction of the building. The east side, the one with the scaffolding, was misplaced, turning by 90 degrees in the plan and giving to the arch an “L” shape. The result was always the same, even when the accuracy of the alignment was set to the highest value. Additionally, the overlap on that side seemed to be correct. Looking carefully at the images of the east and north sides, the maxi-screens on the scaffoldings were broadcasting the same images with the same timing both on the north and east edges of the arch (Figure 8).

**Figure 9:**
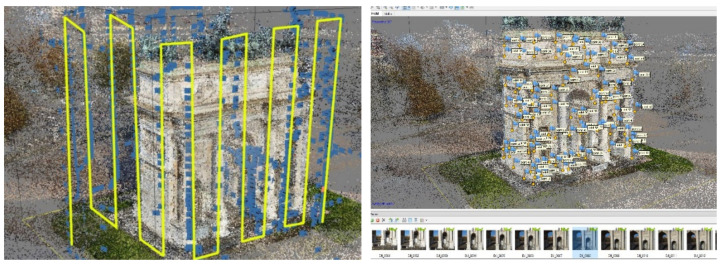
On the left, the regular manual path followed during the flights; on the right, the natural measured points.

**Figure 10:**
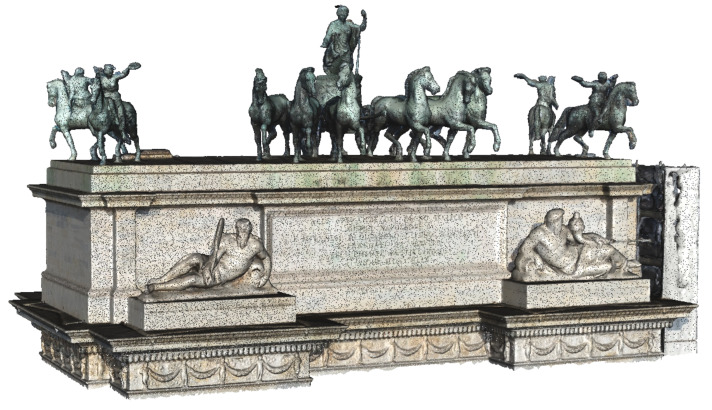
UAV photogrammetric dense cloud of the upper part of the arch.

The areas of the photos with the screens were masked directly in the software, and the alignment then gave proper results. Then, the natural points were checked and placed on the images, and the topographic measurements were imported into the project (Figure 9). After the optimisation process, the mean error on the points measured by the operator is equal to 2 cm, so the model can be used to sample measurements compatible with the tolerance of 1:50 drawing scale (Figure 10). As described in paragraph 5.6, the 3D model of the arch was developed starting from point clouds data in the 3D modelling software McNeel Rhinoceros version 7.

#### 5.3.2. The Statues, Ornaments, and Bass Reliefs

Elaboration of the statues, ornaments and bass reliefs followed the same pipeline of the arch, but the models were computed separately for each decorative element. This elaboration phase aims to obtain the NURBS models to be included in the general model of the arch. Unlike the classical architectonical elements, such as walls, pillars, columns, and friezes that simple geometries can describe, the statues and decorations require a different approach to generate the NURBS models. The classical elements are modelled, extracting sections and elevations directly from the point clouds, and the study of the geometries is supported by historical and design drawings.

**Figure 11:**
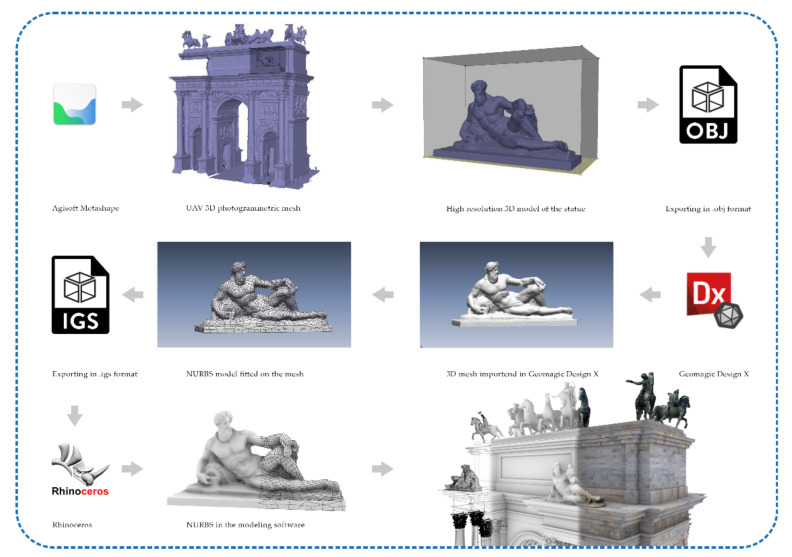
The workflow to generate the NURBS geometries of the statues and the decorations.

On the other hand, it is impossible to adopt the same approach for completely free forms elements such as statues. Obviously, these elements could not be neglected in the restitution phase of the model, but it was not possible to shape them by extracting generating features and patching lines. The solution adopted was to perform the transformation with reverse engineering software, such as Geomagic Design X v 5.1. This step requires correcting typical mesh errors: auto intersecting, non-manifold, crossing, redundant, tangled, reversed faces, small tunnels, and duplicated vertices. They would cause bad results after the auto surfacing command that fits NURBS on the targeted meshes. For this reason, the elaboration of these last elements considered the following steps (Figure 11):

Agisoft Metashape

- Duplicating the original UAV chunk;
- Resizing of the bounding box around each statue and bass relief;
- Elaboration of the depth maps at the highest resolution;
- Generation of the meshes using as source the depth maps;
- Export of the meshes in .obj format.

Geomagic Design X

- Import the .obj files;
- Fixing the topological errors of the meshes;
- Creating a watertight mesh;
- Auto fitting the NURBS geometries on the meshes;
- Export the meshes in .igs format.

McNeel Rhinoceros

- Import the .igs file without scaling or moving the single object.

### 5.4. Terrestrial Data Elaboration

**Figure 12:**
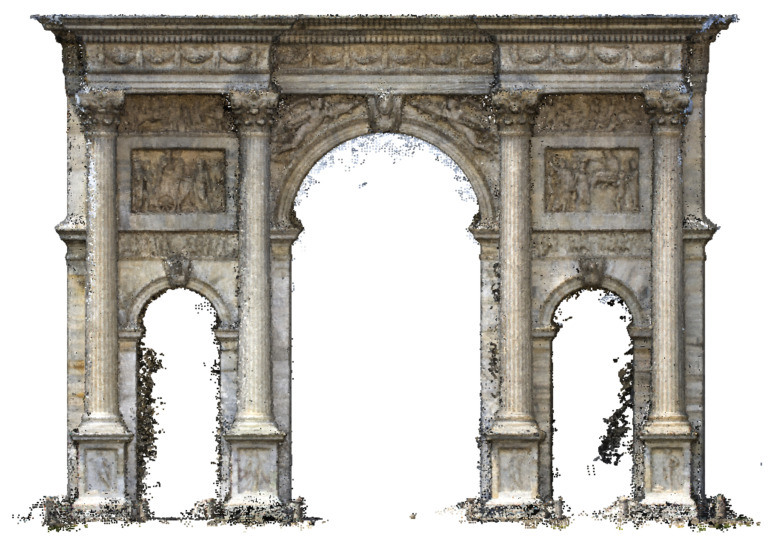
North elevation of the arch, terrestrial photogrammetric point cloud.

The terrestrial dataset comprises 229 images, and the elaboration phase followed the same pipeline of the UAV photogrammetric project, giving results in terms of accuracy comparable to those described above. This elaboration aimed to produce a dense point cloud of the lower part of the architecture to be merged with the dense point cloud and meshes coming from the UAV dataset elaboration (Figure 12).

### 5.5. Data Merging

**Figure 13:**
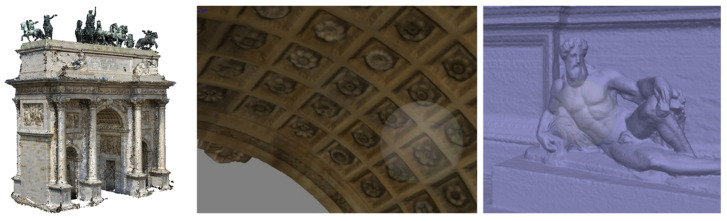
On the left, final merged 3D point cloud. In the centre, decorative apparatus recorded from the terrestrial photogrammetric survey; on the right, 3D model of one of the statues on the top of the arch acquired with the UAV survey.

The two datasets did not share the coordinates because each one has its own local reference system measured with the total station. To place the two models in the same position, some manual points of the same architectonical elements were collected on both projects. The points are well distributed on three of the four elevations. Then, a new Agisoft Metashape project was created to append the terrestrial and UAV chunks. The former one was aligned to the latter using the architectonic points and fixing the scale of both models. After cleaning the overlapping parts, the two models were merged, saving the best geometries of each one, i.e., the bottom part of the terrestrial survey and the upper part of the UAV survey (Figure 13).

### 5.6. HBIM Generation: From Mesh-Textured Models to NURBS Models and Heritage Building Information Modelling

Thanks to the integration of primary and secondary data sources, the digitisation process of the monument was able to benefit from point clouds coming from aerial photogrammetry and many documentations and historical drawings capable of communicating the constructive logic of the building. As anticipated in paragraph 5.3, thanks to the integrated use of point clouds and textured mesh models, it was possible to lay the appropriate foundations for defining a method capable of representing any type of shape in BIM logic. In particular, the use of GOG 9 and 10 made it possible to extract geometric primitives, slices, and wireframe models directly from point clouds and mesh models from aerial and terrestrial photogrammetry.

**Figure 14:**
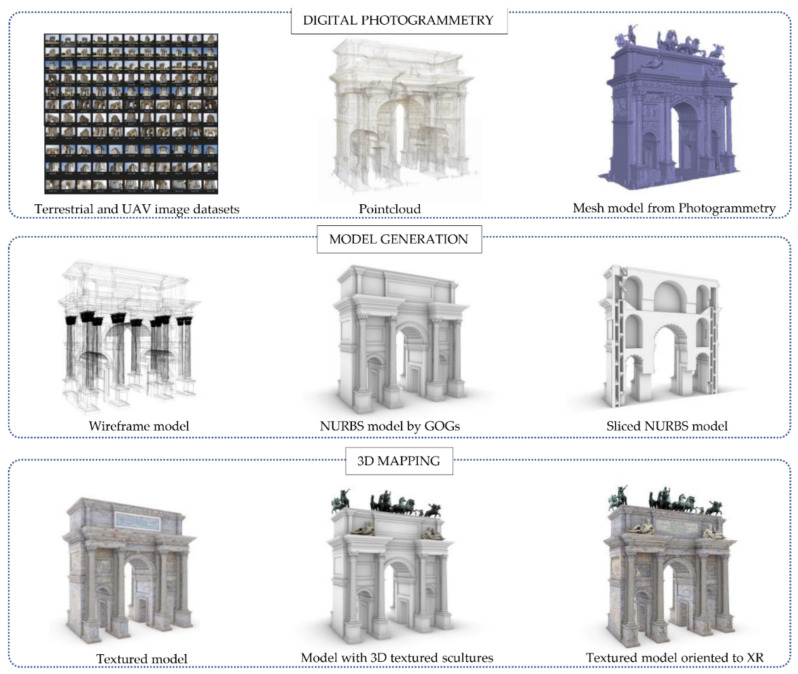
The scan-to-NURBS process applied to the Arco della Pace in Milan.

Figure 14 shows the multi-step approach, moving from simple points in space or mesh polygons to a NURBS model capable of corresponding to the surveyed reality. Thanks to NURBS modelling, it has been possible to create mathematical models capable of going beyond the limits imposed by BIM applications, which are still characterised by a very limited number of 2D representation and 3D modelling tools.

**Figure 15:**
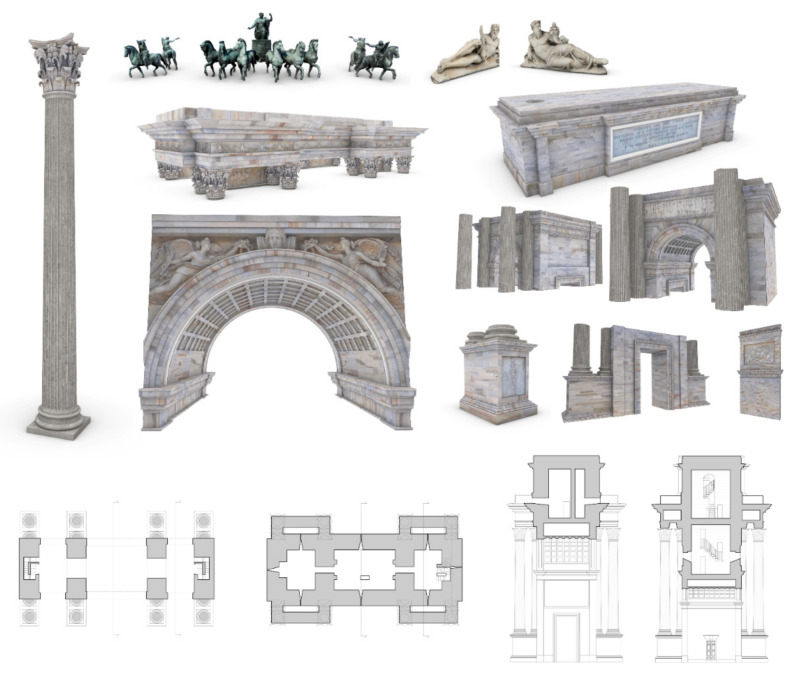
The HBIM objects of the research case study and the main 2D drawings extracted from the HBIM project browser.

The second step allowed the transformation of NURBS models into HBIM objects capable of communicating high levels of information (Figure 15). Unlike newly constructed buildings, historic buildings require a 3D mapping phase capable of communicating their unique architectural, structural, material, and decorative features. Each historic building is a world unto itself, where generic materials and textures enucleated in the BIM libraries do not allow an appropriate representation of the structure, and consequently the mapping and sharing of information is not always truthful. For this reason, aerial photogrammetry has enabled a 3D mapping phase capable of communicating the integrity of the materials for each individual digitised element, from the coffered vaults, to the pillars, up to the sculptures and low reliefs. The latter also required the transmissibility of material information and the development of new BIM parameters able to tell the story represented through textual descriptions. On the other hand, BIM applications are not easily usable by non-expert users in 3D digitalisation. For this reason, the method envisaged the development of advanced XR environments capable of reaching a wider audience and consequently enhancing the communication levels of digital models for students, virtual tourists, and other forms of users.

Once the various NURBS objects were transformed into BIM parametric objects, thanks to the verification of the grade of accuracy (GOA), it was possible to communicate the reliability of each element created thanks to point set deviation analysis. In particular, thanks to an automatic verification system (AVS), the standard deviation between point clouds and BIM objects was calculated [23,56,57]. The value reached for every single element allowed us to define a GOA of about 1–2 mm. Consequently, the development of HBIM parameters capable of communicating this value within the propriety window of each object has allowed the user to identify the GOA, the LOD obtained, the scale of representation and the creation of schedules and databases able to accurately compute numerical quantities such as area and volume and subsequently define the materials and restoration phases useful for the conservation of the monument over time.

### 5.7. Synchronising HBIM Models with XR Development Platforms: The Virtual Visual Storytelling of the Arco della Pace in Milan

**Figure 16:**
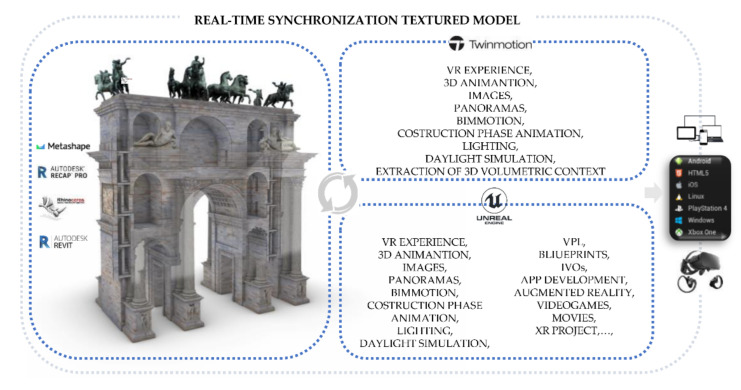
XR development approach applied to the research case study.

The level of interactivity achieved was, however, limited to a small circle of experts. Consequently, the passage from the information mapping phase to the information sharing phase led the authors to develop new interactivity levels, exploring the latest generation techniques and tools and defining a development process capable of immersing any type of user in XR environments (Figure 16).

In this specific context, the study and understanding of the development techniques of XR environments made it possible to test and define a process based on the use of open-source platforms such as Unreal Engine, unity and Twinmotion. Unlike the scan-to-BIM-to-XR methods already consolidated in recent years, the main added value of the method was found thanks to the possibility of the synchronisation of multiple modelling software and XR development platforms, avoiding interruptions and development discontinuity between opening and closing one software to another. Thanks to the development of new add-ins, functionalities integrated into software architecture, in addition to exponentially reducing XR development times, it has been possible to define a workflow that can also be applied to experts in the construction and virtual museum sector [58], who do not always possess computer skills capable of increasing the level of interactivity of their digital models.

A second benefit of the proposed method derives from a synchronised mapping technique between Autodesk Revit, McNeel Rhinoceros and XR software such as Twinmotion and Unreal engine. In particular, the method required the use of many images from aerial photogrammetry. It has been found that the main 3D mapping techniques in modelling software and BIM platforms involve the use of decals. Decals are non-repeating textures that are applied to the surface of an object with a given projection. Decals are textures placed directly on the specified area of one or more objects. Decals are used to change a limited part of an object colour. Decals should be thought of as a single specific texture, rather than side-by-side textures, as they are when used in a material. This is an easy way to apply single images or similar textures to objects without going through complex texture mapping operations.

**Figure 17:**
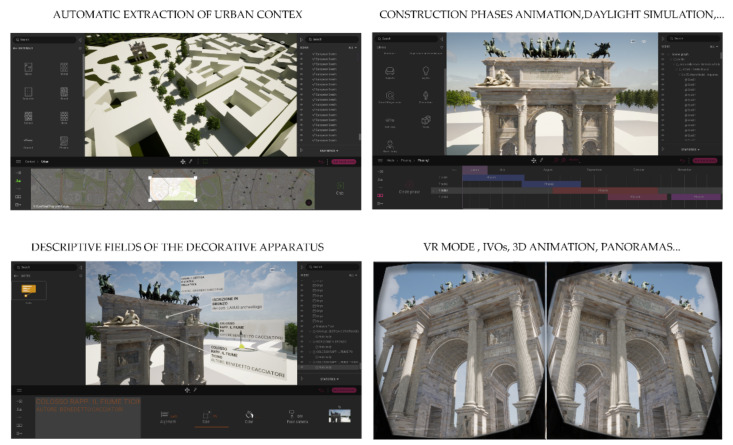
VR project developed in Twinmotion: the development was based on the real-time synchronisation between NURBS modelling software and multiple BIM platforms (McNeel Rhinoceros and Autodesk Revit). It allows the user to obtain different interactive tools, such as the automatic extraction of the urban context, descriptive fields of the decorative apparatus, 3D animation, VR mode, construction phase animation and other types of output and interaction.

On the other hand, it was found that the mode of synchronisation of digital models with XR platforms could not include this technique capable of correctly representing the uniqueness of the monument. Furthermore, thanks to the exhibition developments in computer graphics and the development of the Twinmotion software, it was possible to reduce the phase of graphic post-production and 3D mapping, directly using textures pre-processed in NURBS modelling software and BIM platforms such as McNeel Rhinoceros, Autodesk Revit and Graphisoft Archicad. It has been found that Twinmotion is a Real-Time Rendering software for Architecture, which has recently reached its best performing version; in fact, with just a few clicks, the last version (2021) allows you to connect with the major CAD software existing on the market, such as Archicad, Revit, Sketchup Pro and Rhinoceros; thanks to Twinmotion 2021, it was possible to animate a large number of interactive virtual objects (IVOs), improving and optimising the textures; accelerating the rendering process, which is reduced to a few seconds; and using objects present in the software library or imported from the web (Figure 17).

**Figure 18:**
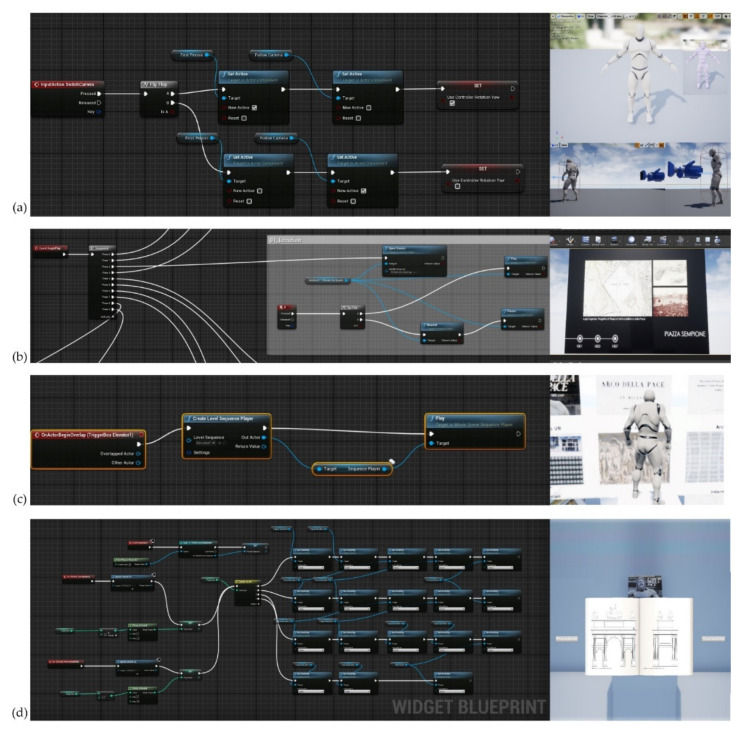
The main Blueprints developed for the XR project: (**a**) change of XR mode between first and third person, (**b**) animation 3D, (**c**) interactive elements for vertical translation, (**d**) interactive book consultation.

For these reasons, the 3D mapping phase had to rely on the definitions of specific textures and the reworking of graphic parameters associated with every single element created. Assuming that a texture can be applied to the surface of a 3D model to add colour, a coating or other details such as gloss, reflectivity or transparency, the problem of representing a texture in 3D rendering can be solved by UV mapping. U and V are the texture coordinates corresponding to X and Y. Think of U as the direction that goes from one side to the other of a quadrangular sheet. Think of V as the other direction, the one that goes from top to bottom. UV texture mapping is used whenever you apply an image to a material and then apply that material to a model. Texture mapping properties manage texture map projections for selected surfaces, polysurfaces, and meshes, representing a 2D image on a 3D model. The mapping transforms a 2D source image into an image buffer called texture. Finally, the last phase of the process involved enhancing the levels of interactivity achieved in the implementation phase. Figure 18 shows the main blueprints developed for the Arco della Pace XR project.

The IT implementation phase envisaged specific blueprints such as Level Blueprints and Blueprint Classes, Blueprint Macros and Blueprint Interfaces. These blueprints contain the scripts necessary for the game level to react to the player’s input with objects that animate, emit sounds, and change their composition based on the player’s actions. The structure is also designed so that the creator of levels can reuse the same Blueprints for the same or slightly different functions, without having to redo the same work for each game element.

One type of Blueprint class is Construction Script, which kicks into action when an actor is set in the game level or updated. It serves to manage the changes that the actor needs when certain events occur. The Blueprint Visual Scripting system in Unreal Engine is a complete gameplay scripting system based on the concept of using a node-based interface to create gameplay elements from within Unreal Editor. Blueprints is the visual scripting system inside Unreal Engine 4 and is a fast way to start prototyping your game. Instead of writing code line by line, you do everything visually: drag and drop nodes, set their properties in a UI, and drag wires to connect Object-Oriented (OO) classes or objects in the engine as with many common scripting languages. This system is highly flexible and powerful as it allows designers to use virtually the full range of concepts and tools generally only available to programmers.

In addition, Blueprint-specific markup, available in Unreal Engine C++ implementation, enables programmers to create baseline systems that designers can extend.

The latter, once developed, made it possible to create static objects without any level of interaction coming from the scan-to-BIM process described here, passing from simple static meshes to IVOs capable of responding to user input. Moreover, thanks to a process of defining the virtual-visual story telling of the monument (VVS) it was possible to tell, as well as with high levels of interactivity, even with virtual rooms where every single decorative apparatus, sculpture and low relief has been digitised and inserted in a museum itinerary. Thus, with any type of device (mobile, tablet, VR headset or PC), the user can remotely explore the intangible values reported in the XR project and become aware of the historical, cultural background of the monument and Milan.

The VVS was developed based on the following XR environments: two interactive menus, in which, thanks to the implementation of specific trigger boxes and blueprints, it was possible to migrate the user in first or third person to new levels. The bass relief, for example, are initially placed at a distance from their true position; when you approach the model, they move until they reach their respective position on the Arc. The Trigger Box is one of the actors that can be activated and cause events in the level; they are used to trigger events in response to interaction with them within the level. The Trigger Box is a trigger that can be placed in the project by dragging it into the layer. In the project, the event that activates the Trigger Box is the overlap of the avatar with the trigger. The various levels identified made it possible to define a progressive narration of the monument: from general information and multimedia files that describe the city of Milan, the square, and the cultural, historical, and geographical context of the monument up to a second menu where rooms were created dedicated to a museum display of the decorative apparatus. After placing the Trigger Boxes, the nodes are developed within the Blueprint level. The second interactive element, the video file, is played on a static mesh with the media source asset file.

The steps carried out were the following:

- creation of the Movies folder within the Content in which to place the video in .mp4;
- through File Media Source and Media Player, the video is associated with the project within the Content. The video resource is generated accordingly;
- creation of the Mesh, that is, the surface on which the video will be visible;
- Simply by dragging the video asset onto the mesh, you relate the video to the surface;
- development of nodes within the Blueprint level so that the video is played on the mesh starting from the start of the virtual experience. This happens automatically, but only through a keyboard command, “P”, which allows you to start and pause the multimedia content.

Inside some ideal rooms, books have been inserted, referring to the bibliography essential to the project. These were made as Widgets, again based on Blueprint. The books are then displayed only after pressing a key, so you can choose independently whether to display them and when. Once opened, the manual is displayed in the foreground. Within the Designer Mode, the actual book was created, inserting one page at a time and the respective drawings. Subsequently, by switching to Graph editing Mode, the nodes necessary for the animation of the book were created. One part concerns the activation of the Widget, through a button, the respective link to the avatar, and finally the sounds of the pages when they are browsed. The other part instead concerns how and when the pages are visible. Everything is based on links between the pages so that the one you are on is the only one visible while the others remain invisible. Therefore, everything is based on a system of switches that allow these settings to be changed once the buttons next to the book have been pressed to move to the next or previous page.

Consequently, the user can immerse himself and understand the story represented through IVOs, digital archives, interactive books and multimedia files that have different kinds of content. For most of the sections, the method of creating the project based on the “Blueprint third-person template” makes it possible to change it in person thanks to creating a Blueprint that allows a quick exchange to take place by pressing a button. In this way, it is possible to better view the various sections by passing from a more immersive view, such as the first-person view, to one that allows you to better compare and understand the dimensions in relation to the height of the avatar. This was carried out within the avatar’s Blueprint, in which the second camera was set at face height to view the scene in first person.

**Figure 19:**
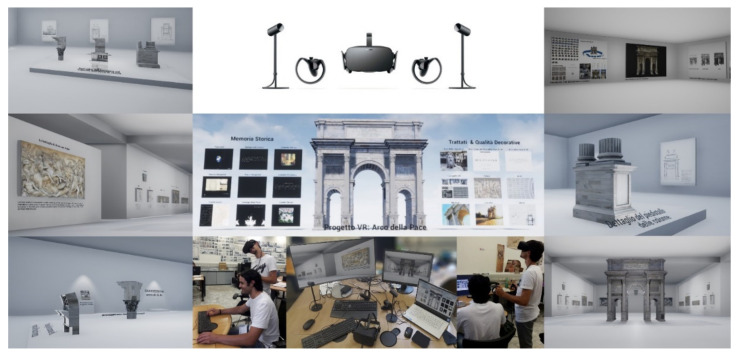
The main section of the Virtual-Visual Story telling of the monument: from virtual museum to interactive virtual objects (IVOs).

The project is divided into levels: in the main one, there is the model of the arch, with the interactive menu to the left and right of the latter. The other levels house the in-depth rooms, which can be reached from the main room (Figure 19). The following Place Actors have been placed inside each one: AtmosphericFog, BP_Sky_Sphere, and ExponentialHeighFog, which complete the preliminary light setting of the levels. To import the files that make up the model, the settings that regulate the collision for each solid object can be changed so that the solids and voids are correctly processed.

Finally, the complete version of the XR project was geared towards the integrated use of VR, the Oculus Ref., a virtual reality device that allows high-quality immersive vision. It consists of a viewer, audio headphones, sensors and two controllers. The sensors are used to track the user’s movements, while the controllers allow you to interface with the experience in a more interactive way and manipulate the objects within the VR project using your hands in a rather realistic way. Through sensors, tracking allows the user to look around in the virtual environment exactly as if it were in the real world. This system allows for the most natural interaction possible, improving the sensation of immersion. Within the Unreal Engine program, it is possible to convert the project and make it compatible with vision through Oculus Rift. To do this, it is necessary to change the display settings by selecting VR Preview, expanding the menu next to the Play icon, and thus starting playback with the viewer and controller. At the end of the elaborations, the models of the 8 statues (3 lying men, 4 knights and a chariot with 6 horses) were placed in their correct position. It was not necessary to move or scale them because we were careful not to change the reference system during each import and export phase.

### 5.8. From HBIM Models and IVOs to Augmented Reality

The proposed method has made it possible to create an immersive environment in all respects. The final user can interact with many IVOs and discover information, from historical-cultural content to precise information such as the descriptions of each low relief, sculpture, etc. Thanks to the in-depth historical research that allowed for the implementation of the VR project and the virtual museum of the monument itself, a further implementation phase was conducted with the ultimate goal of achieving an alternative form of human-computer interaction.

In recent years, several studies have developed applications capable of creating, setting up and sharing AR objects. It has been found that unlike VR, which reproduces the real world to create digital spaces, AR understands and includes the real world, superimposing virtual images on real environments, spaces and images.

In particular, AR was considered by the authors to be a suitable solution for several reasons:

- use of IVOs and HBIM objects for different purposes concerning VR,
- addition of new levels of information, in real-time and with a high rate of interaction using mobile devices of any kind, including wearable technologies,
- superimposition of multimedia information on what you are watching on any display (text, images, live or animated films),
- access to an AR system via the web through devices equipped with GPS, a web camera and an internet connection,
- use and accessibility is within reach of any type of user (expert, professional, students, virtual tourists and on-site tourists) through web apps that can be easily downloaded via the app store,
- creation of a personal account that can be implemented over time,
- ability to view objects and their information in a targeted manner, avoiding having to access the general model of the arch and discriminate between other objects,
- avoid the installation of particular software applications and use expensive digital devices,
- sharing of the model through simple links.

**Figure 20:**
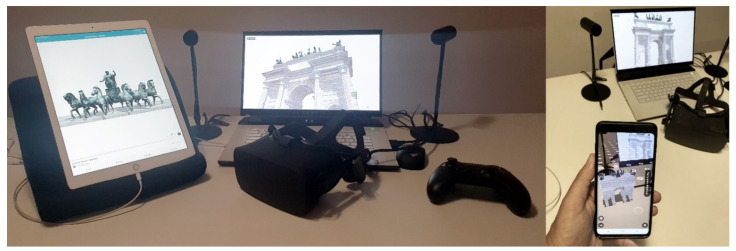
AR implementation: the web-based AR platform of the Arco della Pace, Milan, Italy.

Figure 20 shows the web-based AR library developed for the Arco della Pace. Each object is easily navigable and viewable in AR mode. The associated information has been selected to reach the user in a targeted manner (on-site or remotely) with precise and concise descriptive texts, thus providing a cognitive approach to the decorative schema of the monument without great effort.

### 5.9. Critical Analysis of the Proposed Workflow: Pros and Cons Found during the Implementation Process

The method proposes a continuum that, starting from the real world, leads to a completely virtual interactive world, representing “possible worlds” to create a “sense of presence and interaction” in the user. The relationship between IVOs, information and the user thus becomes the first factor of scientific investigation. The established relationships must be deeply tested in this specific context, guaranteeing the best possible experience from different perspectives. Theoretical assumptions on the use of virtual realities have been dealt with in-depth by the technologist Giti Javidi [59], who has identified positive theoretical correlations between constructivism and virtual learning environments. Through these developments, it has been suggested that, using the XR project, hundreds of specific objectives can be pursued by different means (texts, discussions, videos, software, podcasts, etc.), and the use of VR is just one of them. Pedagogist Veronica Pantelidis expressed her opinion on the conditions that recommend VR, especially for learning and teaching. Based on the points reported in her analysis [60,61], some considerations founded by the authors during the final development phase are here reported. The development of an XR environment can be useful and used effectively:

- if the simulation as an alternative to the real environment allows for greater, more intuitive and faster learning,
- if the interaction with a model is more motivating than the interaction with reality,
- if you travel, costs or logistical difficulties in reaching the site make virtual reality more convenient,
- if the experience of creating a simulated environment or model is important to achieve learning objectives,
- if the visualisation of information and its manipulation using graphic symbols and the latest generation tools can be more easily understood, making the imperceptible perceptible,
- if it is necessary to develop a participatory environment that can only exist if generated with a computer,
- if it is necessary to give disabled people the opportunity to experiment, which they could not do otherwise,

The conditions that advise against the use of virtual reality in teaching are the following:

- whether the “real” learning environment is available and accessible,
- if interaction with real humans, professionals, tutors, teachers, students is necessary,
- if the use of a virtual environment can be physically or emotionally damaging,
- if the use of a virtual environment can provoke a simulation so convincing as to lead some participants to confuse the model with reality,
- if virtual reality is too expensive to justify in light of the expected results.

In addition to these pros and cons of a general nature and applicable to possible future developments in this field, the Arco della Pace case study has highlighted how the XR has become an innovative tool thanks to its multisensory and engaging nature, satisfying the principles of active learning. In fact, immersive virtual experiences have favoured the sense of presence and embodiment, both key factors capable of promoting learning and knowledge of intangible values such as the historical and cultural background represented in the decorative apparatus of the monument.

Learning these values became an active process in which the person builds his knowledge by extracting meanings from interactions with the surrounding virtual world. Thanks to interacting and extracting meanings from the objects surrounding him, the user creates mental models to understand reality. Consequently, the proposed virtual path became a dynamic process in which the person is the protagonist and active participant in the learning process. In turn, it has fostered an emotionally positive experience of involvement, promoting the onset and maintenance of high levels of attention and concentration.

On the other hand, it is also essential to consider the consequences of using virtual reality in health. In fact, some studies have found numerous problems related to what is called “cybersickness”, or symptoms of motion sickness due to diving. Participants sometimes reported experiencing headaches, nausea, disorientation and vision problems. Accordingly, the levels of interactivity developed had to deal with requirements that made them capable of not incurring the issues recently reported by the first manufacturers of gaming platforms. Consequently, the XR projects proposed in this study had to avoid VR sickness (dizziness, nausea, disorientation, sweating, and others). One of the main factors that can affect this is the framerate dropping too low. In Table 5, the recommended framerates for several of the VR headsets that Unreal Engine supports are reported:

Finally, VR environments require the utmost attention from the user (who is immersed in a reconstructed environment within which he can move and interact only “digitally”), making the technology inadequate for interaction. In this context, AR makes it possible to integrate the experience perfectly into the daily interactions that users have in the real world, facilitating collaboration between teams located in different places or accelerating digital learning, design and innovation processes. For these reasons, the development of a web-based AR library has made it possible to increase the usefulness of digital models, defining a new way of sharing information and objects simultaneously. On the other hand, the development of the library itself inevitably had to face specific requirements that led to a reduction in terms of LOD and LOI, such as

- the formats to be used (FBX, OBJ),
- the limited size of the shared models in terms of bytes (50,100,200 MB),
- the reduction of the result of the textures associated with the models (value to be considered in the general size of the AR object),
- compatibility with web browsers (desktop and mobile), and
- navigation and controls (Interface, Orbit Mode and First-Person Mode)

## 6. Discussion and Conclusions

An XR project has been developed to share the model and the related tangible and intangible values. Computer vision and imaging processing allow authors to improve the information mapping and sharing of the scan-to-HBIM process, creating novel XR environments containing the history of the monuments, high-resolution models of the statuary and the decorative apparatus and interactive virtual objects (IVO). New exchange formats, new game engine platforms, and visual scripting were used to complete the architectural study of such an important monument in the context of Milan. Right from the start, the work carried out aimed to certify how XR relates to the memory and custody of the built heritage, in particular the Arch of Peace in Milan, which in recent years has had less and less maintenance and restorations. Thanks to technological evolution, the virtual experience has become indispensable for the enjoyment of the architectural, artistic and cultural heritage to an increasingly large audience. In fact, this method shows how integration between the scan-to-BIM process, HBIM and XR allows users, in addition to painstakingly and faithfully recomposing any type of object, in this architectural case, to implement the knowledge of our built heritage. XR sees an infinite application in many fields, and its use in the context of cultural heritage and built heritage has an enormous development prospect. Moreover, Italy contains an extensive cultural heritage within its territory, and the possible digitisation of this capital is one of the primary purposes of researchers, scholars, and experts in the sector. As previously mentioned, a fundamental role is recognised in continuous technological development, which contributes to improving virtual reality experiences, making the generative process and the subsequent governmental act even more immediate.

## Figures and Tables

**Table 1:** List of regulations issued over the years in Italy and Europe.

Document	Edition	Date	No. of Pages
Italian Regulation (UAV)	First Edition	16/12/2013	21
	Second Edition	16/07/2015	37
	First Amendment	21/12/2015	37
	Second Amendment	22/12/2016	37
	Third Amendment	24/03/2017	37
	Fourth Amendment	21/05/2018	37
European Regulation (not effective in Italy)	First Edition	24/05/2019	27
Italian Regulation (UAV)	Third Edition	11/11/2019	37
	First Amendment	14/07/2020	37
European Regulation (effective in Italy)	First Edition	31/12/2020	27
Italian Regulation (UAS-IT)	First Edition	04/01/2021	20

**Table 2:** Open category scheme after the 1st of January 2023, https://www.easa.europa.eu/domains/civil-drones-rpas/open-category-civil-drones (accessed on 14 July 2021).

UAS		OPERATION		DRONE OPERATOR/PILOT		
Class	Maximum Take Off Mass (MTOM)	Subcategory	Operational Restrictions	Drone Operator Registration	Remote Pilot Competence	Remote Pilot Minimum Age
Privately built	<250 g	A1 (can also fly in subcategory A3)	May fly over uninvolved people (should be avoided when possible) No flying over assemblies of people	No, unless camera/sensor on board and drone is not a toy	No training needed	No minimum age
C0					Read user manual	16, no minimum age if drone is a toy
C1	<900 g		No flying expected over uninvolved people (if it happens, should be minimised) No flying over assemblies of people	Yes	Read user manual Complete online training Pass online theoretical exam	16
C2	<4 kg	A2 (can also fly in subcategory A3)	No flying over uninvolved people Keep horizontal distance of 30 m from uninvolved people (this can be reduced to 5 m if low speed function is activated)	Yes	Read user manual Complete online training Pass online theoretical exam Conduct and declare a self-practical training Pass a written exam at a recognised entity	16
C3	<25 kg	A3	Do not fly near people Fly outside of urban areas (150 m distance)	Yes	Read user manual Complete online training Pass online theoretical exam	16
C4						
Privately built						

**Table 3:** Specification of DJI Mavic Mini.

DJI Mavic Mini—Specs	
Sensor size (pixel)	4000 × 3000
Sensor size (mm)	6.48 × 4.86
Pixel size (mm)	0.00162
Focal length (mm)	4.49
Flight time (min)	28

**Table 4:** Specification of Canon EOS 1100D.

Canon EOS 1100D—Specs	
Sensor size (pixel)	4272 × 2848
Sensor size (mm)	22.2 × 14.7
Pixel size (mm)	0.00534
Focal length (mm)	18

**Table 5:** Specification to avoid VR sickness.

Device	Frame Per Second (FPS)
Vive	90
Gear VR	60
PSVR	Variable up to 120
Rift Retail	90
DK1	0
DK 2	75

## Data Availability

Data will be available upon request.
